# Early Decision Indicators for Foot-and-Mouth Disease Outbreaks in Non-Endemic Countries

**DOI:** 10.3389/fvets.2016.00109

**Published:** 2016-11-30

**Authors:** Michael G. Garner, Iain J. East, Mark A. Stevenson, Robert L. Sanson, Thomas G. Rawdon, Richard A. Bradhurst, Sharon E. Roche, Pham Van Ha, Tom Kompas

**Affiliations:** ^1^Animal Health Policy Branch, Department of Agriculture and Water Resources, Canberra, ACT, Australia; ^2^Faculty of Veterinary and Agricultural Sciences, University of Melbourne, Parkville, VIC, Australia; ^3^AsureQuality Limited, Palmerston, New Zealand; ^4^Investigation and Diagnostic Centre and Response Directorate, Ministry for Primary Industries, Wellington, New Zealand; ^5^Centre of Excellence for Biosecurity Risk Analysis, University of Melbourne, Parkville, VIC, Australia; ^6^Crawford School of Public Policy, Australian National University, Acton, ACT, Australia

**Keywords:** FMD, early decision indicators, vaccination, simulation models, decision-support, regression analysis

## Abstract

Disease managers face many challenges when deciding on the most effective control strategy to manage an outbreak of foot-and-mouth disease (FMD). Decisions have to be made under conditions of uncertainty and where the situation is continually evolving. In addition, resources for control are often limited. A modeling study was carried out to identify characteristics measurable during the early phase of a FMD outbreak that might be useful as predictors of the total number of infected places, outbreak duration, and the total area under control (AUC). The study involved two modeling platforms in two countries (Australia and New Zealand) and encompassed a large number of incursion scenarios. Linear regression, classification and regression tree, and boosted regression tree analyses were used to quantify the predictive value of a set of parameters on three outcome variables of interest: the total number of infected places, outbreak duration, and the total AUC. The number of infected premises (IPs), number of pending culls, AUC, estimated dissemination ratio, and cattle density around the index herd at days 7, 14, and 21 following first detection were associated with each of the outcome variables. Regression models for the size of the AUC had the highest predictive value (*R*^2^ = 0.51–0.9) followed by the number of IPs (*R*^2^ = 0.3–0.75) and outbreak duration (*R*^2^ = 0.28–0.57). Predictability improved at later time points in the outbreak. Predictive regression models using various cut-points at day 14 to define small and large outbreaks had positive predictive values of 0.85–0.98 and negative predictive values of 0.52–0.91, with 79–97% of outbreaks correctly classified. On the strict assumption that each of the simulation models used in this study provide a realistic indication of the spread of FMD in animal populations. Our conclusion is that relatively simple metrics available early in a control program can be used to indicate the likely magnitude of an FMD outbreak under Australian and New Zealand conditions.

## Introduction

Disease managers are faced with a number of challenges when deciding on the most effective disease control strategy to implement in an exotic animal disease outbreak. Foot-and-mouth disease (FMD) is particularly challenging given its wide range of host species, potential for rapid spread, and serious socio-economic consequences. For countries such as Australia and New Zealand, FMD represents the most serious threat to their livestock industries. A recent study estimated the 2013 value of total direct economic loses over 10 years for a large multi-state outbreak of FMD in Australia at USD 47 billion (1). Animal products constitute a significant proportion of New Zealand exports, and the provisional results of recent modeling of the economic impacts of a large FMD outbreak in New Zealand have estimated net 2014 GDP losses over an 8-year period to be between USD 13 and 17 billion (2). Consequently, Australia and New Zealand invest considerable resources in preparedness and planning for emergency animal disease outbreaks, including maintaining vaccine banks for FMD. Despite recent changes to contingency plans to recognize that vaccination could be an important component of an FMD control program, it is unclear how or when vaccination should be used, and if it is used, how vaccinated animals should be managed once an outbreak has been resolved.

Modeling studies carried out in Australia (3–5) and overseas (6–8) have shown that vaccination is effective in reducing the duration and/or size of FMD outbreaks in situations where disease is widespread, where there is a high rate of spread or the resources for stamping out are limited. Reports suggest that early vaccination may have been beneficial in eradicating the disease earlier than was the case with recent FMD outbreaks in Korea (9) and Japan (10). Thus, vaccination is increasingly being recognized as a potential useful tool to assist in containing and eradicating FMD outbreaks in countries where the disease is not endemic. However, while vaccination may contribute to earlier eradication of the disease, it will be associated with additional costs – keeping vaccinated animals in the population will delay the period until FMD-free status is regained under current World Organization for Animal Health guidelines (11) and add additional complexity to post-outbreak surveillance programs. These issues are of particular concern for countries with significant exports of livestock and livestock products because, under current conditions, the use of vaccination and the presence of FMD vaccinated animals in the population could be expected to cause significant market access difficulties.

From a planning and management perspective, it would be useful to have access to decision support tools that take into account the information that would be available to disease managers early in an outbreak to provide an indication of the potential severity of the outbreak that could ensue. This would enable decisions on specific measures like vaccination to be made at a time when they are likely to be most effective.

McLaws and Ribble (12) documented the relationship between the interval (in days) from incursion to detection and epidemic size [expressed as the total number of infected premises (IPs)] for 24 FMD outbreaks in non-endemic countries that occurred between 1992 and 2003. They did not find a direct relationship between time to detection and total number of IPs or total animals culled for disease control, concluding that the movement of animals through markets was the most critical factor contributing to large outbreaks. Sarandopoulos (13) conducted a review of 125 FMD epidemics in non-endemic temperate countries reported to the OIE between January 1, 2005 and December 31, 2013 to identify associations between epidemic size/duration and early outbreak explanatory variables. The explanatory variables assessed in this study included susceptible animal densities, weather conditions at the time of detection, the number of IPs detected in the first 7 days, and the size of the area under control (AUC) at 7 days (based on a convex hull calculation). In total, ten candidate explanatory variables were tested for their association with epidemic size and duration using a zero-inflated negative binomial regression model. Cattle density, pig density, and the number of IPs at day 7 post-detection were all positively associated with epidemic size while increased average temperature in the month of detection was associated with “smaller” outbreaks.

Using data from the outbreak of FMD that occurred in the UK in 2001, first fortnight incidence (FFI), i.e., the cumulative number of new FMD-IPs found in the first 2 weeks of the response, was found to be a useful predictor of the size and duration of outbreaks at the regional and national scale (14, 15). The larger the number of detected herds within the first 2 weeks, the higher the risk of the large outbreak. Halasa et al. (16) extended the approach of Hutber et al. to incorporate the first fortnight spatial spread (FFS) as well as FFI (which they renamed first fortnight outbreaks – FFO, since a true incidence rate is not actually calculated) – in a simple decision tool using simulated FMD outbreaks. In terms of outcome, in addition to the number of IPs and outbreak duration, they also considered the size of the AUC and costs. Halasa and colleagues found good correlations between FFO and FFS and all of the outcome variables, indicating that both FFO and FFS have the potential as predictors of epidemic outcomes. They also found that the type of index herd was a significant predictor of epidemic outcome.

The combined work of Hutber et al. (15), Halasa et al. (16), and Sarandopoulos (13) indicates that information available early in an outbreak can be used to make inferences about the potential severity of an FMD outbreak and could perhaps be incorporated into decision support tools. However, one of the concerns is that FFO and FFS are quite simple parameters that are likely to be sensitive to outbreak management response, in particular the effectiveness of the surveillance/reporting system. For example, while a low FFO may be indicative of a limited spread and small number of infected places, it could also be indicative of the adequacy of resources to undertake surveillance and tracing. In addition, based on the work of McLaws and Ribble (12) and Sarandopoulos (13), other factors such as animal densities at the location of the index premises and involvement of animal markets may also be important.

With this background, this study was undertaken to identify characteristics measurable during the early phase of a FMD outbreak that might be useful as predictors of the severity of an FMD epidemic (expressed as the total number of infected places, outbreak duration, and the total AUC). The study also aimed to assess how robust findings were across different incursion scenarios and between different production and management systems. A key point is that in this study simulation models of FMD were used to generate a series of outbreaks listing incident infected places over time and geographical space. Regression approaches were then used to identify characteristics measurable during the early phase of a simulated outbreak that might be useful as predictors of the total number of infected places, outbreak duration, and the total AUC predicted by each simulation model. The inferences drawn from this study are dependent on the strict assumption that each of the simulation models used in this study provide a realistic indication of the spread of FMD in animal populations.

## Materials and Methods

A modeling study was undertaken to test a range of explanatory variables as predictors of the total number of infected places, outbreak duration, and the total AUC in an FMD outbreak. The study involved two countries (Australia and New Zealand) and two modeling platforms. Linear regression, classification and regression tree (CART), and boosted regression tree (BRT) analyses were used to assess the association between putative explanatory variables and the three outcome variables.

### Disease Models

The Australian Animal Disease Spread Model [AADIS (17, 18)] is a hybrid model that simulates the spread and control of FMD in livestock populations at a national scale. AADIS uses the herd as the epidemiological unit of interest and models the spread of disease both within and between herds. Spread of disease within a herd is modeled through a deterministic equation-based model, and between-herd spread is modeled with a spatially explicit stochastic agent-based model. There are five discrete spread pathways in the between-herd model: direct animal movements, local spread (infection of farms within close geographical proximity by unspecified means), indirect contact (*via* contaminated equipment, people, or animal products), animal movements *via* saleyards, and windborne spread.

The model incorporates the attributes and spatial locations of individual farms, saleyards, weather stations, local government areas, and various other features of the environment. For FMD control, AADIS is configured to support the range of mitigation strategies described in Australia’s contingency plans for FMD (19) with the effectiveness of these measures dependent on available resources (4).

InterSpread Plus [ISP (20)] is a spatial and stochastic simulation model of infectious disease in domestic animal populations. ISP is a state-transition model meaning that the epidemiological units of interest (farm locations) exist in either the susceptible, infected, or not-at-risk state at any given time. Similar to AADIS, ISP uses a series of user-defined parameters to define the spread of an infectious agent from one farm location to another through local spread, windborne spread, and direct and indirect contacts. Updated movement parameters are informed by findings from recent livestock movement studies in New Zealand (21, 22). Control measures, such as depopulation, vaccination, and movement restrictions, in addition to varying disease surveillance intensity can be simulated, with the ability to carry out each of these activities subject to user-defined resource constraints, similar to the AADIS model.

### Study Design

**Figure 1:**
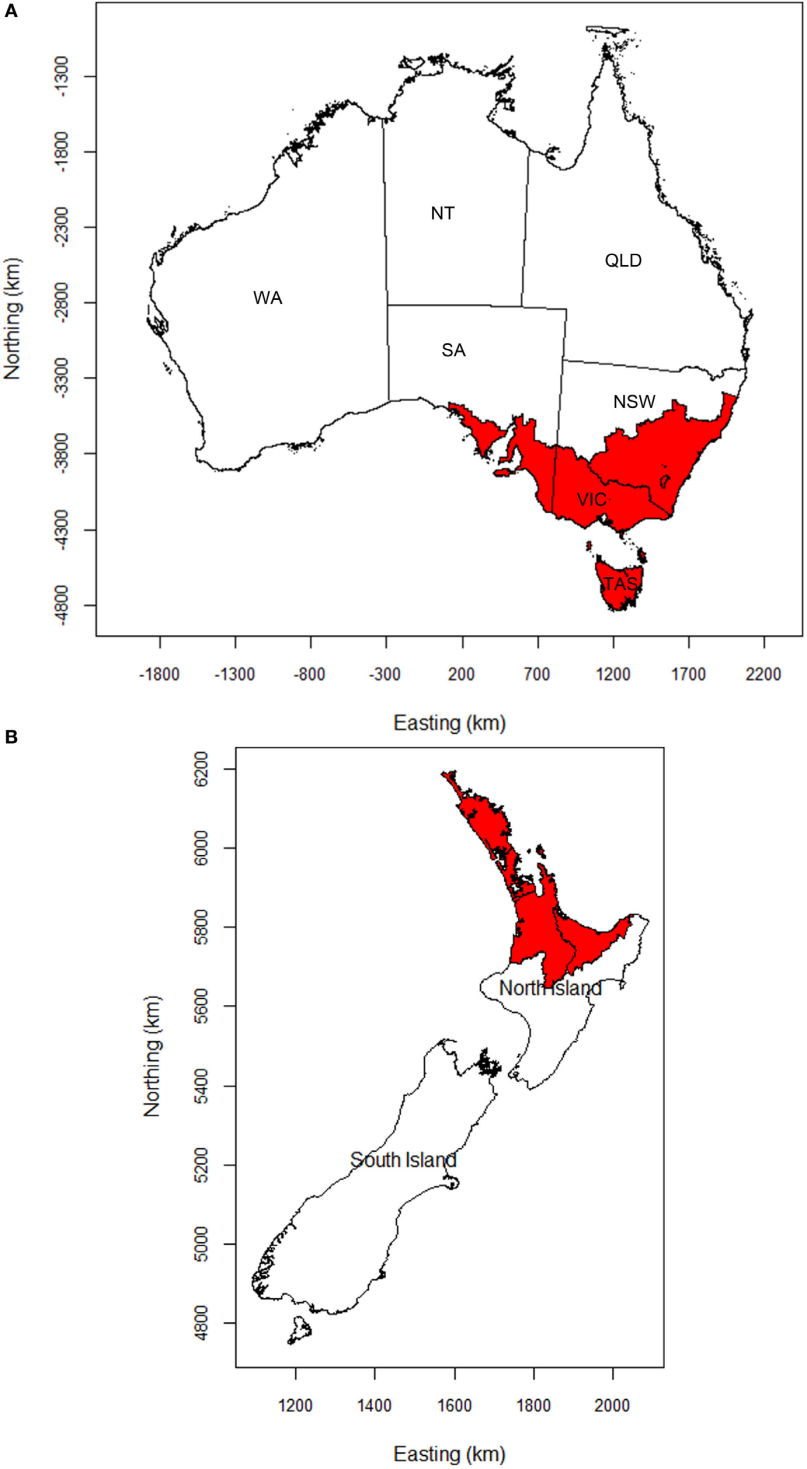
**Maps of (A) Australia and (B) New Zealand showing the areas (shaded), in which FMD outbreaks were initiated**. NSW, New South Wales; VIC, Victoria; TAS, Tasmania; SA, South Australia; WA, Western Australia; NT, Northern Territory; QLD, Queensland.

Epidemics of FMD in Australia and New Zealand were simulated using the AADIS and ISP models, respectively. A total of 10,000 FMD outbreak simulations were carried out using each model. For each simulation, FMD was introduced into a single livestock farm selected at random within assessed high risk areas for FMD. For Australia, the study area was the whole country, with initial seeding of infection confined to south eastern Australia (Figure 1A). South eastern Australia comprises the states of Victoria and Tasmania and parts of New South Wales and South Australia. This area contains a mix of farming enterprises. It is the center of Australia’s dairy production and is considered a higher risk area for introduction, establishment, and spread of FMD (23).

The study area for New Zealand comprised the whole of mainland New Zealand, incorporating the North and South Islands. Initial seeding of infection was confined to the Auckland mega-region (Auckland and its three neighboring regional council areas, Figure 1B) as it is assumed that the most likely introduction scenario for FMD into New Zealand would involve people or contaminated products seeding infection into livestock in this area. The Auckland mega-region has the largest international air and sea ports. Furthermore, yachts visiting the country are more likely to make landfall in the north.

The following assumptions were used for the Australian and New Zealand FMD models. The time from incursion to first detection was probabilistically determined based on farming systems and expected disease reporting rates in the two countries. For Australia, data on the daily probability of detection and the delay from incursion to first detection were sourced from Martin et al. (24). For New Zealand, data on the daily probability of detection were sourced from Murray and Sanson (25). Outbreak control was based on application of animal movement controls, enhanced surveillance, tracing, and stamping out (i.e., destruction, disposal, and decontamination) on detected IPs. These were applied according to each country’s FMD response plan (19, 26). Resources for disease control were based on each country’s estimates of expected resources (5). Each model run ended when disease was eradicated or after 1 year, whichever occurred first.

### Explanatory Variables

Three time points (days 7, 14, and 21 after first detection) were selected, and candidate explanatory variables based on data that would be available to disease managers at these time points were collated: (1) outbreak location: farm and animal densities around the site of first detection; (2) the involvement of markets/saleyards; (3) measures of the geographic distribution of IPs, as measured by the AUC, and the number of discrete disease clusters; (4) measures of temporal spread, as measured by the number of IPs reported, and the number of traced premises identified; (5) the rate of disease spread, as measured using the estimated dissemination ratio (EDR), calculated using the methods described by Miller (27) and Morris et al. (28); and (6) adequacy of resources available for control. A description of each of the candidate explanatory variables is provided in Table 1.

**Table 1:** **Explanatory variables tested**.

Metric/parameter	Details
Location characteristics – farm density, animal density (cattle, sheep, pig), human density at first detected farm site	Calculated for 5 km × 5 km cell centered on the index farm
Markets/saleyards involvement	Any IP infection(s) *via* saleyard pathway recorded as a 0/1 for days 7, 14, and 21
Size of area under control (AUC)	Based on a dissolved polygon constructed around IPs using a 10 km buffer at days 7, 14, and 21
Number of clusters	The number of non-contiguous polygons using a 10 km radius buffer around IPs at days 7, 14, and 21
Number of IPs	The number of IPs reported at days 7, 14, 21
Number of traced premises	The cumulative number of backward and forward traced premises at days 7, 14, and 21
Estimated dissemination ratio (EDR)	Four-day EDR calculated at days 14 and 21
Resources	Number of premises awaiting destruction at days 7, 14, and 21

For each simulated outbreak, we defined three outcome variables: the total number of IPs, outbreak duration (defined as the number of days from first detection until the date on which the last IP was culled), and the total AUC (in km^2^). Modeling results for each country were analyzed separately.

### Statistical Methods

#### Linear Regression

The Stata/IC statistical package (29) was used for all linear regression analyses. Datasets were imported into Stata and the three outcome variables and each of the explanatory variables checked for normality and log transformed, where necessary, to minimize problems due to non-normality and heteroscedasticity of model residuals (30). Scatterplots of each of the log transformed outcome variables and each of the log transformed candidate explanatory variables were made and the association between variable pairs assessed by superimposing a Lowess-smoothed curve on each plot. After log transformation, all relationships were linear or near linear. Subsequent analyses used two modeling techniques: (1) linear regression modeling using robust estimates to account for non-normally distributed dependent variables (31) and (2) negative binomial regression. It was considered appropriate to use linear regression techniques because the methodology is robust to violations of the requirement for normally distributed dependent variables if the number of observations is large (32, 33). The outputs of the two regression models were similar, so only the results of the linear regressions are presented. The linear regression was preferred because a small proportion of values had excessively large residuals in the negative binomial models.

Candidate explanatory variables were initially tested for unconditional associations with each of the three outcome variables. Explanatory variables that were associated with the outcome variables with *P* < 0.20 were selected for inclusion in the initial multiple regression models. The initial multiple regression model was then reduced step-wise by removing the explanatory variable with the highest *P*_Wald_ value. This process was repeated until all remaining explanatory variables had *P*_Wald_ < 0.05. After the most parsimonious model was developed, all excluded explanatory variables were reassessed by adding them individually back into the model. All biologically plausible, first-order interaction terms were tested, one at a time and retained in the model if the *P*_Wald_ < 0.05 (no interaction terms were retained). The extent of confounding was assessed using the variance inflation factor. No significant confounding was observed in the final models presented.

We acknowledge that the outcome variables measured on a given EDI day (i.e., days 7, 14, and 21 post-detection), which were used as explanatory variables in each model were correlated with their corresponding outcome variable. To investigate this issue further, alternative models were developed where the outcome variable was expressed as (for example) the total number of IPs − IPs identified up to day 14. Using this approach, we identified no substantial differences in the final set of explanatory variables included in the model and the direction and magnitude of the adjusted measure of association between each explanatory variable and the outcome were essentially the same. For this reason, and also to allow our findings to be compared with previous studies (16), we elected to use the total number of infected places, total outbreak duration, and the total AUC as outcome variables in each of the models presented.

While the explanatory variables that remained in each of the Australian and New Zealand regression models differed, those with the most explanatory power (that is, those with the highest beta weight values) were present in both of the country models. For parsimony, a simpler regression model was built using only the explanatory variables that were common to both the Australian and New Zealand models with little or no loss of explanatory power (see Results).

For the linear regression models, the *R*^2^ value is reported as a measure of the goodness of fit of the model. Based on the regression coefficients estimated for the explanatory variables included in each of the three regression models for each country, predictions of the total number of infected places, outbreak duration, and the total AUC were computed. Several cutpoints (e.g., more or less than 20 IPs) were then arbitrarily selected to divide the model iterations into large and small outbreaks. Two by two contingency tables were constructed to compare the regression model estimates with the actual values of the (classified) outcome variables. These data were then used to calculate negative and positive predictive values for the day 14 model estimates using standard techniques (34).

#### Regression Trees

Acknowledging the possibility of non-linear relationships between the explanatory variables and the three outcome variables used in this study, we used CART and BRT analyses as an alternative approach for identifying associations in these data. CART analysis involves recursively partitioning an outcome variable into two parts based on the value of a given predictor variable that best splits the data. A complete CART returns a “tree” with multiple splits, depicted as branches. Predictor variables and their split points are chosen to optimize a given goodness-of-fit criterion, such as minimizing the residual sum of squares (for continuous data). CART analysis is mathematically identical to some multivariable regression techniques, but presents the results in a way that is easily understood by non-technical audiences.

In contrast to CART, a BRT analysis generates a large number of regression trees based on random samples of the data (35). A BRT model returns a list of predictor variables used to create the splits in each of the trees computed using the randomly sampled data. A relative weight is then calculated for each predictor variable by computing the average number of times the variable was chosen for splitting weighted by the squared improvement to the model from each split and scaled to sum to 100. Larger weights indicate a stronger influence between an explanatory variable and the outcome. The BRT analysis requires the analyst to specify the learning rate and tree complexity. Learning rate controls how much each tree contributes to the model as it develops. In general, smaller learning rates result in better predictions than larger learning rates. Tree complexity sets the number of interactions fitted in the model: a tree complexity of two allows for two-way interactions, three allows for three-way interactions, and so on.

Classification and regression tree analyses were carried out for each of the three outcome variables for the Australian and New Zealand data using the rpart package (36) implemented in R version 3.3.1 (37). The BRT analyses were carried out using the dismo package (38) in R.

## Results

Of the 10,000 outbreaks that were simulated, FMD did not establish (there was no spread from the seed herd) in 3210 simulations in Australia and 1180 simulations in New Zealand. These simulations were excluded from subsequent analyses. Descriptive statistics of the simulated outbreaks and explanatory variables for Australia and New Zealand are shown in Tables 2 and 3, respectively. Descriptive statistics of the outcome variables for the AADIS (Australia) and ISP (New Zealand) models (that is, the total number of infected places, outbreak duration and the total AUC) are shown in Table 4.

**Table 2:** **Descriptive statistics of explanatory variables from the AADIS model of foot-and-mouth disease**.

Variable	*n*	Mean (SD)	Median (Q1, Q3)	Min, max
**Day 7**				
IPs	6790	4 (3)	3 (2–6)	1, 24
AUC (km^2^)	6790	734 (579)	459 (344–927)	300, 4242
Clusters	6790	2 (2)	1 (1–3)	1, 14
IPs per km	6790	0 (0)	0 (0–0)	0, 0
Traces	6790	3 (3)	2 (0–4)	0, 30
**Day 14**				
IPs	6790	9 (10)	5 (3–10)	1, 82
EDR	6790	0.55 (0.89)	0 (0–1)	0, 15
AUC (km^2^)	6790	1168 (1282)	651 (357–1394)	300, 10,980
Clusters	6790	3 (3)	2 (1–4)	1, 28
IPs per km	6790	0 (0)	0 (0–0)	0, 0
Traces	6790	6 (7)	3 (1–8)	0, 97
**Day 21**				
IPs	6790	12 (16)	5 (3–13)	1, 148
EDR	6790	0.42 (0.77)	0 (0–0.78)	0, 9
AUC (km^2^)	6790	1297 (1564)	667 (364–1543)	300, 16,270
Clusters	6790	3 (4)	2 (1–4)	1, 34
IPs per km	6790	0 (0)	0 (0–0)	0, 0
Traces	6790	8 (12)	4 (1–9)	0, 147
**Others**				
Cattle density^a^	6790	49 (83)	28 (9–62)	0, 1644
Sheep density^b^	6790	120 (131)	82 (18–176)	0, 1615
Pig density^c^	6790	32 (100)	0 (0–13)	0, 946
Human density^d^	6790	23 (135)	3 (1–8)	0, 3725

*AUC, area under control; EDR, estimated dissemination ratio*.

*^a^Cattle density: number of cattle/km^2^*.

*^b^Sheep density: number of sheep/km^2^*.

*^c^Pig density: number of pigs/km^2^*.

*^d^Human density: number of humans/km^2^*.

**Table 3:** **Descriptive statistics of explanatory variables from the InterSpread Plus model of foot-and-mouth disease**.

Variable	*n*	Mean (SD)	Median (Q1, Q3)	Min, max
**Day 7**				
IPs	8784	9 (10)	6 (3–12)	1, 141
AUC (km^2^)	8784	934 (623)	739 (452–1216)	314, 5856
Clusters	8784	2 (1)	1 (1–2)	1, 10
IPs per km	8784	0 (0)	0 (0–0)	0, 0
Traces	8784	12 (12)	8 (4–16)	0, 113
**Day 14**				
IPs	8784	15 (18)	9 (4–20)	1, 218
EDR	8784	0.69 (0.93)	0.5 (0–1)	0, 19
AUC (km^2^)	8784	1169 (830)	928 (576–1553)	314, 7368
Clusters	8784	2 (1)	1 (1–2)	1, 10
IPs per km	8784	0 (0)	0 (0–0)	0, 0
Traces	8784	16 (16)	11 (5–22)	0, 148
**Day 21**				
IPs	8784	20 (23)	11 (5–25)	1, 255
EDR	8784	0.62 (1.05)	0.2 (0–1.0)	0, 20
AUC (km^2^)	8784	1287 (930)	1021 (617–1716)	314, 8310
Clusters	8784	2 (1)	1 (1–2)	1, 9
IPs per km	8784	0 (0)	0 (0–0)	0, 0
Traces	8784	18 (18)	12 (5–24)	0, 165
**Others**				
Cattle density^a^	8784	166 (84)	152 (104–217)	0, 570
Sheep density^b^	8784	86 (79)	70 (24–122)	0, 893
Pig density^c^	8784	5 (24)	0 (0–1)	0, 349
Human density^d^	8784	891 (2162)	273 (153–653)	4, 24,048

*AUC, area under control (km^2^); EDR, estimated dissemination ratio*.

*^a^Cattle density: number of cattle/km^2^*.

*^b^Sheep density: number of sheep/km^2^*.

*^c^Pig density: number of pigs/km^2^*.

*^d^Human density: number of humans/km^2^*.

**Table 4:** **Descriptive statistics of the three outcome variables from the AADIS model of FMD in Australia and the InterSpread Plus model of FMD in New Zealand**.

Model – outcome variable	*n*	Mean (SD)	Median (Q1, Q3)	Min, max
**AADIS**				
Total number of IPs	6790	22 (51)	6 (3–16)	2, 844
Outbreak duration	6790	53 (38)	43 (30–61)	16, 365
Area under control	6790	1523 (2136)	680 (368–1669)	300, 29,953
**InterSpread Plus**				
Total number of IPs	8784	32 (46)	15 (5–39)	2, 424
Outbreak duration	8784	52 (28)	43 (31–64)	21, 263
Area under control	8784	1542 (1220)	1176 (636–2110)	316, 12,815

For the New Zealand (ISP) simulations, the area used for seeding FMD outbreaks had a substantially higher density of cattle (median of 152 head/km^2^) than the areas where FMD was seeded for the Australian (AADIS) simulations (median of 28 head/km^2^).

Compared with the FMD outbreaks simulated by AADIS, ISP simulated relatively high numbers of IPs during the early phase of each epidemic. The median number of IPs on days 7, 14, and 21 for ISP was 6, 9, and 11 (respectively) compared with 3, 5, and 5 for AADIS (Tables 2 and 3). Similarly, the number of traces generated by ISP in the early phase of each epidemic was higher than those generated by AADIS. The median number of traces generated by days 7, 14, and 21 for ISP was 8, 11, and 12 (respectively) compared with 2, 3, and 4 for AADIS. There are three possible explanations for these findings: (1) differences in characteristics of the countries and/or study regions and incursion scenarios used for each model; (2) differences in model parameterization, resulting in different probabilities of farm-to-farm transmission of virus; and (3) differences in model design (in ISP the probabilities of transmission vary according to farm type but not farm size whereas in AADIS both farm size and farm type influence probabilities of transmission).

Outbreak durations for the two models were similar: a median of 43 (minimum 16, maximum 365) days for AADIS compared with a median of 43 (minimum 21, maximum 263) for ISP. The size of the AUC was substantially lower for the AADIS simulations. The median AUC for the AADIS simulations was 680 km^2^ (minimum 300, maximum 29,953) compared with 1176 km^2^ (minimum 316, maximum 12,815) for ISP.

### Linear Regression

Regression coefficients and their standard errors for the linear regression models of the total number of infected places, outbreak duration, and the total AUC for the AADIS and ISP models of FMD are provided in Table 5. Table 6 provides details of the goodness of fit (*R*^2^) for each of the linear regression models developed for Australia and New Zealand. A consistent pattern was observed with the goodness of fit of the models improving from days 7 to 14 to 21 for all outcome variables for both the Australian and New Zealand data sets.

**Table 5:** **Regression coefficients and their standard errors for the multivariable linear regression models of first 14-day predictors of area under control, the total number of infected places, and outbreak duration for the AADIS and InterSpread Plus models of FMD**.

Explanatory variable	Coefficient (SE)	*t*	*P*-value	95% CI
**AADIS – total number of IPs**				
Intercept	−0.02 (0.019)	−0.80	0.421	−0.05 to 0.02
Number of IPs at day 14	1.27 (0.008)	164.51	<0.001	1.25 to 1.28
Pending culls at day 14	0.18 (0.017)	10.64	<0.001	0.15 to 0.22
**AADIS – outbreak duration**				
Intercept	13.87 (0.480)	28.88	<0.001	12.92 to 14.81
Area under control day 14	0.39 (0.008)	51.72	<0.001	0.38 to 0.40
EDR day 14	0.12 (0.009)	14.31	<0.001	0.11 to 0.14
IP density day 14	18.45 (0.721)	25.60	<0.001	17.04 to 19.86
First detected farm type				
Beef intensive	Reference			
Dairy	−0.09 (0.021)	−4.16	<0.001	−0.13 to −0.05
Feedlot	−0.20 (0.020)	−9.98	<0.001	−0.23 to −0.16
Mixed beef-sheep	0.11 (0.018)	6.24	<0.001	0.08 to 0.14
Pigs (large)	−0.50 (0.020)	−25.28	<0.001	−0.54 to −0.46
Pigs (small)	−0.27 (0.017)	−15.66	<0.001	−0.30 to −0.24
Sheep	0.25 (0.019)	12.73	<0.001	0.21 to 0.28
Smallholder	−0.26 (0.048)	−5.44	<0.001	−0.35 to −0.17
**AADIS – area under control**				
Intercept	−0.57 (0.023)	−25.09	<0.001	−0.62 to 10.52
Area under control day 14	1.10 (0.003)	313.84	<0.001	1.09 to 1.11
**InterSpread Plus – total number of IPs**				
Intercept	0.19 (0.016)	11.63	<0.001	0.15 to 0.22
Number of IPs at day 14	1.11 (0.006)	174.93	<0.001	1.10 to 1.12
Pending culls at day 14	0.13 (0.010)	13.22	<0.001	0.11 to 0.15
**InterSpread Plus – outbreak duration**				
Intercept	7.07 (0.173)	40.76	<0.001	6.73 to 7.41
Area under control day 14	0.29 (0.005)	55.24	<0.001	0.28 to 0.30
EDR day 14	0.21 (0.007)	29.62	<0.001	0.19 to 0.22
IP density day 14	7.82 (0.241)	32.36	<0.001	7.34 to 8.29
First detected farm type				
Dairy dry	Reference			
Lifestyle	−0.003 (0.015)	−0.25	0.800	−0.03 to 0.03
Beef-sheep-mixed	−0.042 (0.016)	−2.63	0.009	−0.07 to −0.10
Dairy milking	−0.084 (0.016)	−5.33	<0.001	−0.12 to −0.05
Pig breeding	−0.138 (0.087)	−1.59	0.111	−0.31 to 0.03
Pig fattening	0.232 (0.271)	0.86	0.392	−0.30 to 0.76
**InterSpread Plus – area under control**				
Intercept	−0.28 (0.027)	−10.45	<0.001	−0.33 to −0.23
Area under control day 14	1.07 (0.004)	275.96	<0.001	1.06 to 1.08

*EDR, estimated dissemination ratio*.

**Table 6:** **Goodness-of-fit statistics (*R*^2^) for each of the linear regression models for the total number of infected places, outbreak duration, and area under control using days 7, 14, and 21 explanatory variables for the AADIS and InterSpread Plus models of FMD**.

Model – outcome	Day 7	Day 14	Day 21
**AADIS**			
Total number of IPs	0.84	0.92	0.96
Outbreak duration	0.61	0.71	0.77
Area under control	0.77	0.96	0.98
**InterSpread Plus**			
Total number of IPs	0.73	0.85	0.91
Outbreak duration	0.43	0.58	0.67
Area under control	0.73	0.85	0.91

Positive and negative predictive values for “large” or “small” outbreaks (for the total number of IPs and total AUC) or “short” or “long” outbreaks (for outbreak duration) for AADIS and ISP are shown in Table 7. The proportions of correctly classified outbreaks ranged from 0.88 to 0.97 for AADIS and 0.79 to 0.92 for ISP.

**Table 7:** **Positive and negative predictive values and the proportion of outbreaks correctly classified as large or small (or short or long) using the day 14 linear regression model for the AADIS and InterSpread Plus simulated FMD outbreaks**.

Model – outcome	Cut point^a^	Predictive value		Correctly classified
		Positive	Negative	
**AADIS**				
Total number of IPs	20	0.97	0.83	0.96
Total number of IPs	54	0.97	0.80	0.95
Outbreak duration	54	0.94	0.68	0.88
Outbreak duration	90	0.95	0.62	0.94
Area under control	1000	0.98	0.91	0.96
Area under control	3000	0.98	0.88	0.97
**InterSpread Plus**				
Total number of IPs	20	0.89	0.87	0.88
Total number of IPs	54	0.94	0.77	0.92
Outbreak duration	54	0.85	0.64	0.79
Outbreak duration	90	0.92	0.52	0.91
Area under control	1000	0.94	0.87	0.85
Area under control	3000	0.94	0.79	0.92

*^a^Used to classify outbreak as small or large*.

### Regression Trees

Classification and regression tree analyses were carried out to identify factors associated with the total number of IPs, outbreak duration, and total AUC. Similar to the approach used for the linear regression analyses, three sets of explanatory variables were used: those at day 7 post-detection, day 14 post-detection, and day 21 post-detection. Using these three sets of explanatory variables with each of the three outcome variables and both the Australian and New Zealand data sets resulted in 18 CART analyses in total. BRT models using the same explanatory variables and the same outcome variables were developed using the Australian and New Zealand data.

**Figure 2:**
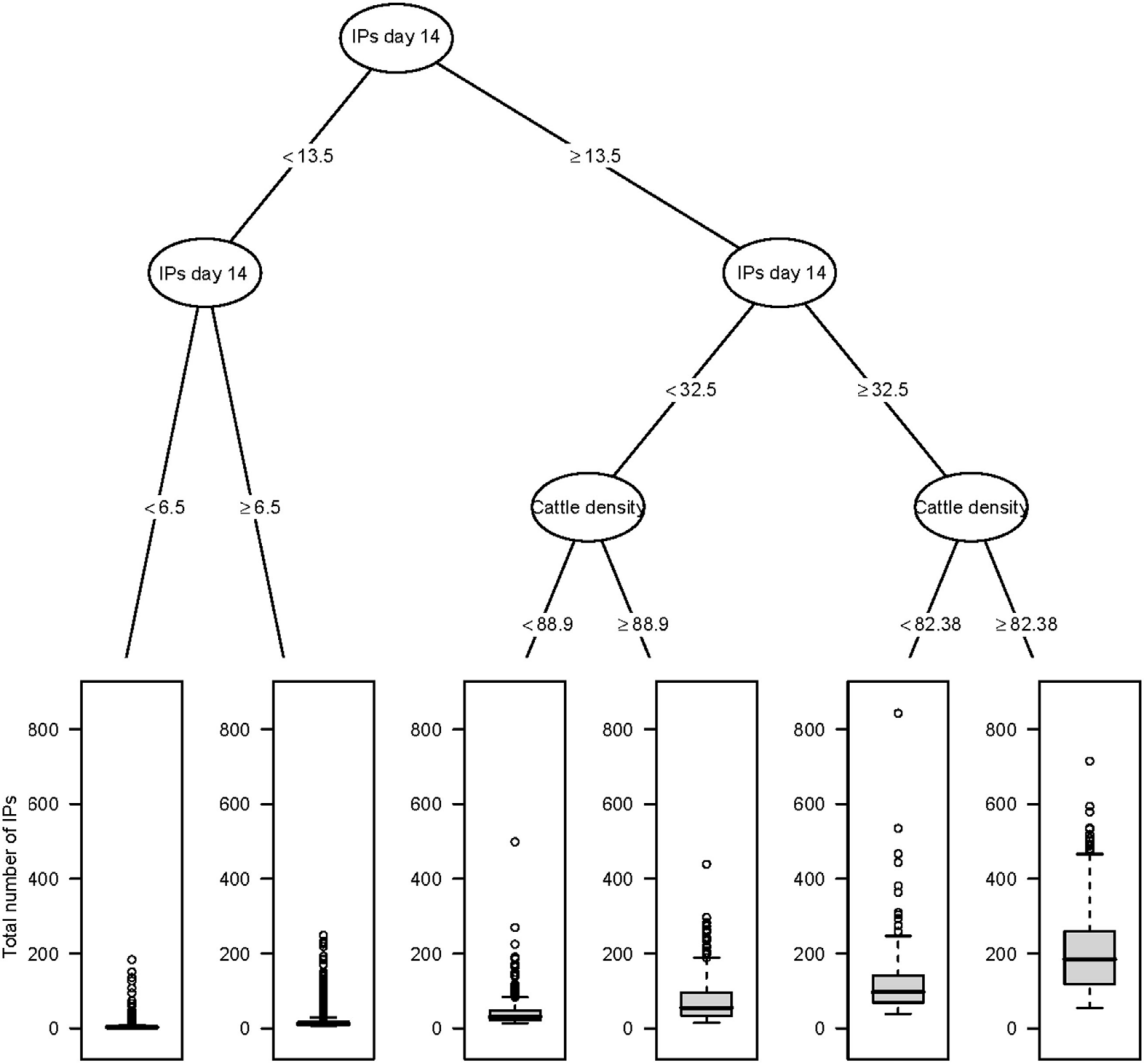
**Classification and regression tree summarizing day 14 post-detection variables predictive of the total number of IPs using AADIS**. The number of IPs identified at day 14 post-detection had the strongest association with the total number of IPs followed by cattle density at the location of the index premise. Relatively large outbreaks were those where there were more than 32 IPs identified by day 14 and where cattle density at the location of the index premise was greater than or equal to 82.38 head/km^2^.

**Figure 3:**
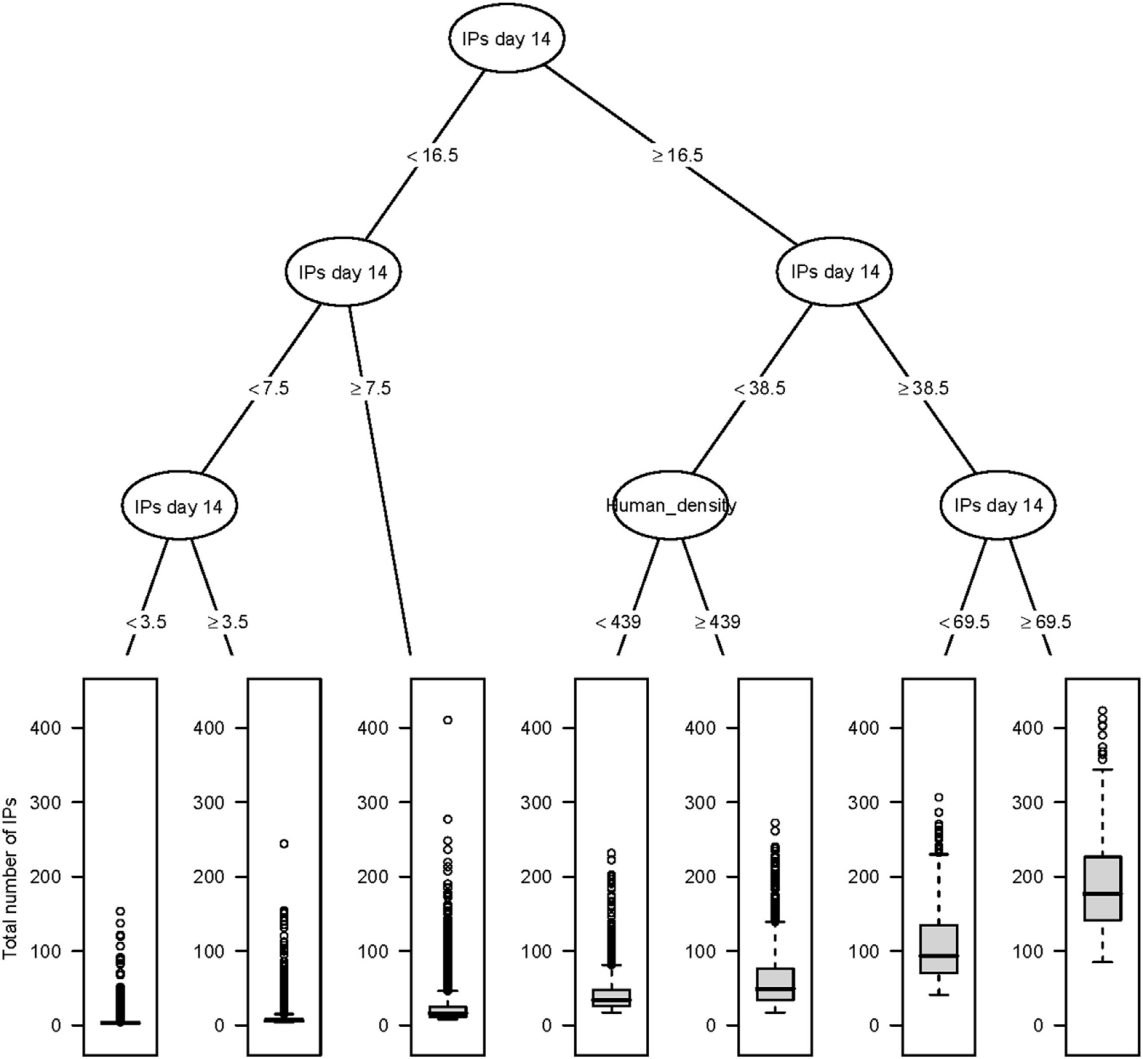
**Classification and regression tree summarizing day 14 post-detection variables predictive of the total number of IPs using InterSpread Plus**. The number of IPs identified at day 14 post-detection had the strongest association with the total number of IPs followed by human population density at the location of the index premise. Relatively large outbreaks were those where there were greater than or equal to 69.5 IPs identified by day 14.

The CART for the predicted total number of IPs using day 14 explanatory variables for the Australian and New Zealand data are shown in Figures 2 and 3, respectively. For both the AADIS and ISP models, the number of IPs at day 14 had the greatest influence on the total number IPs. For the AADIS model, in addition to the number of IPs identified at day 14, the total AUC at day 14 and cattle density influenced the total number of IPs.

The five most influential explanatory variables (and their weights) from the BRT models for the total number of IPs, outbreak duration, and the total AUC for the Australian and New Zealand data are listed in Table 8. Consistent with the CART analyses, for both AADIS and ISP, the number of IPs identified at day 14 was associated with each of the three outcome variables. While for ISP the number of IPs identified at day 14 had the highest weight for each of the three outcomes, the total number of outbreak clusters identified at day 14 had the greatest weight as a predictor of the total AUC for AADIS. For AADIS, the density of cattle was associated with each of the three outcome variables, albeit with a relatively low regression weight in each model (10.2, 15.4, and 0.1 for the total number of IPs, outbreak duration, and the total AUC, respectively).

**Table 8:** **Identified explanatory variables (*n* = 5) and their weights (in brackets) for the boosted regression tree model of first 14 day predictors of area under control, the total number of infected places, and outbreak duration for the AADIS and InterSpread Plus models of FMD**.

Model – outcome	Explanatory variables (weights)
**AADIS**	
Total number of IPs	IPs day 14 (60.8), cattle/km^2^ (10.2), AUC day 14 (9.4), number of traces day 14 (4.0), EDR day 14 (3.1)
Outbreak duration	IPs day 14 (40.7), cattle/km^2^ (15.4), AUC day 14 (9.3), number of pigs/km^2^ (7.6), EDR day 14 (6.2)
Area under control	Number of clusters day 14 (90.1), IPs day 14 (9.6), IPs/km^2^ (0.1), number of traces day 14 (0.1), cattle/km^2^ (0.1)
**InterSpread Plus**	
Total number of IPs	IPs day 14 (81.4), human population density (5.1), EDR day 14 (3.5), IPs/km^2^ day 14 (2.3), cattle/km^2^ (1.7)
Outbreak duration	IPs day 14 (50.4), human population density (14), EDR day 14 (12.1), IPs/km^2^ day 14 (5.4), cattle/km^2^ (4.5)
Area under control	IPs day 14 (39.8), number of traces day 14 (36.3), IPs/km^2^ (11.6), number of clusters day 14 (7.2), EDR day 14 (2.3)

The predictive ability of each of the day 14 boosted regression models was assessed by calculating the positive and negative predictive values for each model (Table 9), similar to the approach taken for the linear regression models. Overall, the BRT models were able to correctly classify simulated outbreaks as either large or small (for the total number of IPs and total AUC) or short or long (for outbreak duration) with the proportion of correctly classified outbreaks ranging from 0.82 to 0.96 for AADIS and 0.77 to 0.93 for ISP. In general, negative predictive values for the BRT models were greater than the positive predictive values.

**Table 9:** **Positive and negative predictive values and the proportion of outbreaks correctly classified as large or small (or short or long) using the day 14 boosted regression tree model for the AADIS and InterSpread Plus simulated FMD outbreaks**.

Model – outcome	Cut point^a^	Predictive value		Correctly classified
		Positive	Negative	
**AADIS**				
Total number of IPs	20	0.79	0.97	0.93
Total number of IPs	54	0.76	0.98	0.96
Outbreak duration	54	0.72	0.87	0.82
Outbreak duration	90	0.70	0.96	0.94
Area under control	1000	0.93	0.96	0.95
Area under control	3000	1.00	0.90	0.90
**InterSpread Plus**				
Total number of IPs	20	0.79	0.91	0.86
Total number of IPs	54	0.74	0.95	0.91
Outbreak duration	54	0.64	0.85	0.77
Outbreak duration	90	0.63	0.92	0.91
Area under control	1000	0.90	0.89	0.90
Area under control	3000	0.81	0.94	0.93

*^a^Used to classify outbreak as small or large*.

## Discussion

During a disease outbreak, decisions on control are often made under significant uncertainty and in conditions that are continually evolving. Resources are often limited and will influence the effectiveness of disease control efforts. Experience overseas suggests that resource and logistical issues are critical considerations when evaluating disease control strategies (39–41). Vaccination is increasingly being recognized as an important tool to assist in containing and eradicating FMD outbreaks (6–8, 42, 43). Vaccination has been shown to be most effective in situations where disease is spreading rapidly or resources are inadequate to maintain effective stamping out (4). A number of studies have shown that vaccination is more beneficial when used early in an outbreak (8, 44, 45).

Although vaccination can be an important tool to control FMD, it will make achieving recognition of FMD-free status more difficult – keeping vaccinated animals in the population will delay the period until FMD-free status is regained under the World Organization for Animal Health (OIE) guidelines and add additional complications to the post-outbreak surveillance program (46). Shifting attitudes to vaccination among the international veterinary community means that it is no longer viewed as a measure of last resort. In Australia and New Zealand, vaccination will be given consideration as a potential additional measure (alongside stamping out) from day one of any FMD eradication response. However, given the complications and costs associated with implementing a vaccination strategy, it would only be used if authorities consider that it would be beneficial in managing the outbreak (19, 26, 47). A decision to vaccinate early in the outbreak may result in situations where it was not actually required and have consequent implications for post-outbreak surveillance, management of vaccinated animals, and regaining FMD-free status and access to markets. Conversely, not using vaccination in some situations may lead to larger and longer outbreaks, increased control costs, and greater on-going impacts on industry and local communities.

Although Australia and New Zealand have developed frameworks to support decision-making on FMD control (19, 48), these are qualitative and subjective. We reason that it would be useful if disease managers could identify early in an outbreak those situations that are likely to progress to “large” outbreaks and for which additional measures like vaccination are likely to be beneficial. In this context, measurable parameters such as the number of IPs, numbers of traced premises and/or farms under surveillance, and estimated rates of spread might be useful indicators of the potential severity of an outbreak.

The overarching aim of this project was to identify factors that could be used to predict the total number of IPs, outbreak duration, and the total AUC. Here, “factors” refers to characteristics of the physical environment in which an FMD incursion first occurs (e.g., farm density, animal density, human population density) or characteristics of the outbreak itself (e.g., the number of IPs reported at a given point in time post first detection). We were particularly interested in how robust the findings were to outbreaks in different settings. For this study, we used a wide range of FMD incursions in terms of location, production systems and seed farm type, and time to first detection (determined probabilistically). These outbreaks were simulated in two countries using two independent modeling platforms.

It is reassuring for animal health authorities that, in both countries, the simulated FMD outbreaks tended to be small and readily able to be contained and eradicated with available resources. For both countries, median outbreak durations were around 6 weeks. This finding assumes that FMD is reported relatively quickly and resources are adequate to implement effective control programs. For Australia, the median time from first introduction to reporting was 17 days (range 9–89), and for New Zealand, the median time to detection was 13 days. A previous Australian study found considerable regional variability in the probability that an individual infected farm would report suspect FMD (24, 49). Recent experience of outbreaks of FMD in non-endemic countries indicate that it can take up to 3 weeks after introduction of the virus to the primary farm before the disease is recognized (40, 50–52). However, early detection does not necessarily mean that an outbreak will be small. A total of 3.4% of the 10,000 outbreaks of FMD in Australia that were simulated in this study had more than 100 IPs and 7.2% of the 10,000 outbreaks lasted longer than 90 days. For New Zealand, there was a 7.2% probability of an outbreak involving more than 100 IPs and an 8.6% probability of an outbreak lasting more than 90 days.

The key objective of this study was to test whether information known or available to disease managers early in an FMD outbreak could be used to predict the severity of the epidemic outcome. Epidemic outcome was defined in terms of the total number of IPs, outbreak duration, and the total geographic AUC. While FFO and FFS have been shown to correlate with epidemic size (16), it was recognized that it would be more useful to consider a broader range of times than just 14 days. Accordingly, three time points were considered: 7, 14, and 21 days into the control program. A range of potential explanatory variables were tested using different analytical approaches, including linear regression, CART, and BRT analyses.

Although there was some variability between the different analyses and between countries, the cumulative number of IPs at specified time points early in the outbreak were consistently found to be strongly associated with the final number of IPs and the duration of an outbreak. It was possible to build relatively simple linear regression models for predicting the magnitude and duration of simulated FMD outbreaks that fitted both the Australian and New Zealand data (see Table 5). *R*^2^ values as a measure of goodness of fit ranged from 0.3 to 0.9 depending on time point, outbreak variable, and country (Table 6). A consistent pattern was observed, with the fit of the models improving from days 7 to 14 to 21 for all dependent variables and for both data sets (Australia and New Zealand). The total AUC had the highest predictability and duration of an outbreak the lowest. In this study, we found that the number of IPs occurring up to a given time point provided the most predictive power for both size (total IPs) and outbreak duration. This confirms previous findings by Hutber et al. (15) and Halasa et al. (16). The AUC at a given time point was most predictive of the total AUC.

These findings were confirmed in the CART and BRT analyses. Consistency between the different approaches helps build confidence that the criteria identified are relevant to response decision-making. CART techniques are a useful alternative as they provide a visual decision tree output that is intuitive and likely to be well received by those not familiar with statistical analysis (see Figures 2 and 3). The tree diagrams produced in a CART analysis are consistent with clinical reasoning used by animal health professionals and can help to structure explanations of prediction. Compared with regression-based approaches, an advantage of a CART analysis is that it can accommodate non-linear relationships between an outcome variable and a set of explanatory variables as well as missing data.

Boosted regression trees have the advantages of being able to handle a range of explanatory variable types, not requiring any data transformations, and being able to account for complex, non-linear relationships (35). BRTs are better-able to describe linear relationships and are more robust in terms of predictive accuracy, although interpretability suffers as a result. CARTs and BRTs are complementary. CARTs are relatively simple and provide readily interpretable output; BRTs are more complex and robust, but with reduced interpretability. The BRTs for both countries had good predictive ability when the total number of IPs was less than 100. When the total number of IPs was greater than 100, the BRT analyses tended to under predict total IP numbers.

Although it is informative to build statistical models to summarize factors influencing outputs from complex simulation models of FMD, for disease managers, the key issue is how this information can be used to support decision-making. From a disease manager’s perspective, it is useful to consider how good the models are at predicting small and large outbreaks. To do this, it is necessary to make some judgment calls about what constitutes a “large outbreak.” It is difficult in advance to reach agreement on what are acceptable benchmarks in terms of eradicating FMD, as this will be influenced by the time and location of an outbreak, availability of resources, etc. Accordingly, we looked at a series of arbitrary “cut points” for classifying outcomes into small and large (or long and short) outbreaks. Model sensitivity, specificity, and positive and negative predictive values were calculated using these cut-points. In general, the linear regression models were very good at predicting when an outbreak would be small or short; the positive predictive values varied from 0.85 to 0.98 meaning that a small outbreak was correctly predicted between 85 and 98% of the time. It should also be noted that having predicted a small outbreak at day 14 (which would probably mean that a decision to vaccinate would not be made), this decision could be revisited at a later time in the outbreak when more information was available. Incorrectly predicting a large outbreak and using vaccine when it is not actually required will have trade implications and increase outbreak costs. The models were less accurate at predicting a large or long outbreak with the negative predictive values for outbreak duration exceeding 90 days being as low as 0.52 for the models of FMD in New Zealand. The negative predictive values for the total number of IPs and the total AUC were better ranging from 0.77 to 0.91 for both AADIS and ISP.

The BRT models were able to correctly classify simulated outbreaks as either large or small with the proportion of correctly classified outbreaks ranging from 0.77 to 0.96. Negative predictive values tended to be higher than the positive predictive values for the BRT models.

In conclusion, this study shows that based on simulated FMD outbreak data relatively simple metrics available at 1–3 weeks into the control program can be used to predict the size of an FMD outbreak under Australian and New Zealand conditions and provide a basis for making decisions on the use of vaccination as a control measure. It should be noted that the simulation modeling analyses carried out for this study focused on introduction of FMD into the areas considered to be at higher risk of disease entry and dissemination in Australia and New Zealand (23). The results need further validation with modeling data generated from other areas of these countries. Finally, it should be recognized that in the absence of FMD outbreaks in Australia and New Zealand, this study has fitted statistical models to simulated, not real outbreak data. Although the modeling teams have been careful to parameterize the respective models as realistically as possible, it is inevitable that assumptions and extrapolations from overseas experience have had to have been made. These considerations need to be taken into account when using the findings from this study.

## Author Contributions

All the authors made substantial contributions to the work, through concept and design (MG, TR, RS, SR, TK, and PH); data generation and modeling (MG, TR, RS, and RB); statistical analyses (IE, MS, and RS); or interpretation (MG, IE, MS, TR, and TK). MG, IE, and MS had prime responsibility for drafting and revision. MG, TR, and TK have approved the final version for publication and are in agreement to be accountable for all aspects of the work in ensuring that questions related to the accuracy or integrity of any part of the work are appropriately investigated and resolved.

## Conflict of Interest Statement

The authors declare that the research was conducted in the absence of any commercial or financial relationships that could be construed as a potential conflict of interest. The reviewer PW and handling Editor declared their shared affiliation, and the handling Editor states that the process nevertheless met the standards of a fair and objective review.
